# Can percolation theory explain the gelation behavior of diblock copolymer worms?†
†Electronic supplementary information (ESI) available: Full experimental details including synthesis and characterisation of diblock copolymers; GPC chromatograms; SIPLI images; digital images of tube inversion test; calculations for effective worm density, worm cross-sectional radius and volume fraction. See DOI: 10.1039/c8sc02406e


**DOI:** 10.1039/c8sc02406e

**Published:** 2018-08-02

**Authors:** Joseph R. Lovett, Matthew J. Derry, Pengcheng Yang, Fiona L. Hatton, Nicholas J. Warren, Patrick W. Fowler, Steven P. Armes

**Affiliations:** a Department of Chemistry , The University of Sheffield , Dainton Building, Brook Hill , Sheffield , South Yorkshire S3 7HF , UK . Email: s.p.armes@sheffield.ac.uk; b School of Chemical and Process Engineering , University of Leeds , Leeds , West Yorkshire LS2 9JT , UK

## Abstract

It is well known that polymerization-induced self-assembly (PISA) offers an efficient synthetic route for the production of highly anisotropic diblock copolymer worms. When prepared in aqueous media, such worms form thermoresponsive free-standing hydrogels that are (i) readily sterilizable, (ii) can act as a 3D matrix for the culture of normal mammalian cells and (iii) can induce stasis in human stem cell colonies. Herein we critically examine the gelation behavior of two types of diblock copolymer worms in terms of recent advances in percolation theory for rigid rods, which explicitly account for the effect of rod length polydispersity. More specifically, we use small-angle X-ray scattering (SAXS) to determine the weight-average worm contour length, *L*_w_, and the mean worm cross-sectional radius, *R*. This approach enables a direct comparison to be made between the theoretical critical worm volume fraction, *φ*_c_, required for gelation and the experimental values indicated by rheological measurements and tube inversion experiments. Given that these diblock copolymer worms are relatively flexible rather than truly rod-like, reasonably good agreement between these two parameters is observed, particularly for shorter, relatively stiff worms. For longer, more flexible worms a proportionality constant of approximately two is required to reconcile theory with experimental values for *φ*_c_. These findings are expected to have important implications for the aqueous gelation behavior exhibited by various other anisotropic nanoparticles, such as cellulose nanocrystals and semicrystalline block copolymer rods, and also fibril-forming small molecule (*e.g.* dipeptide) gelators.

## Abstract 2

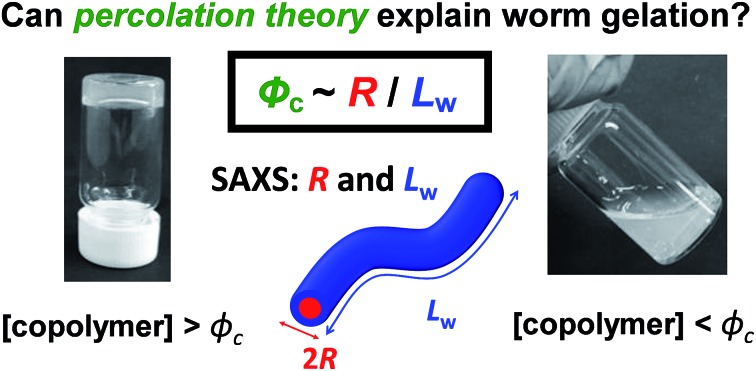
Physical gelation by block copolymer worms can be explained in terms of *multiple inter-worm contacts* using percolation theory, suggesting that *worm entanglements* are irrelevant in this context.

## Introduction

It is well known that certain surfactants (or binary mixtures thereof) can form highly anisotropic worms in aqueous solution.^1^–^4^ These systems have potential applications as thickeners,^5^,^6^ in drag reduction,^7^ and for enhanced oil recovery.^4^,^8^ The ‘living’ nature of these self-healing systems has been demonstrated and sophisticated techniques such as contrast variation neutron scattering have been utilized to characterize their structure.^9^,^10^ Surfactant worms typically exhibit mean contour lengths of the order of 1–10 μm. The concept of worm entanglements as a physical mechanism for gelation has been suggested on the basis of a combination of rheological and theoretical studies.^10^–^12^

Diblock copolymer worm gels have been recognized for almost two decades.^13^ Over the last five years or so, the development of polymerization-induced self-assembly (PISA) has enabled the rational, reproducible synthesis of a wide range of diblock copolymer worm gels directly in water, polar solvents (*e.g.* ethanol) or non-polar solvents (*e.g. n*-alkanes).^14^–^22^ In particular, diblock copolymer worms prepared *via* dispersion polymerization often exhibit thermoresponsive gelation, undergoing a reversible worm-to-sphere morphological transition either on heating in ethanol or *n*-alkanes^18^,^23^,^24^ or on cooling in aqueous solution.^20^,^25^ In each case, this morphological transition appears to be the result of surface plasticization of the core-forming block, which leads to a subtle change in the packing parameter for the diblock copolymer chains.^26^,^27^ Typically, the mean worm width is well-defined, is of the order of a few tens of nm and is dictated by the mean degree of polymerization (DP) of the core-forming block. In contrast, the mean worm length is rather ill-defined and is typically of the order of hundreds of nm. Compared to the dimensions reported for surfactant worms, diblock copolymer worms appear to be too short to account for the observed formation of free-standing gels *via* a worm entanglement mechanism.

Percolation theory has been used for many years to account for the substantial differences in conductivity thresholds observed for many types of conductive particles dispersed in electrically insulating matrices.^28^–^35^ Typically, spheres exhibit a percolation threshold volume fraction of around 0.16,^29^,^30^ whereas highly anisotropic rods (*e.g.* polyaniline needles or carbon nanotubes) form fully-connected conductive networks at significantly lower volume fractions, sometimes below 0.01.^31^–^35^ Recently, percolation theory has been extended to include polydisperse rods exhibiting a wide range of rod lengths,^36^,^37^ which is often the case encountered experimentally. More specifically, for cylindrical rods with a high aspect ratio (*i.e.* length/width ratio), Chatterjee^36^ has used mean field theory to show that the critical volume fraction for the percolation threshold, *φ*_c_, can be estimated using eqn (1):1
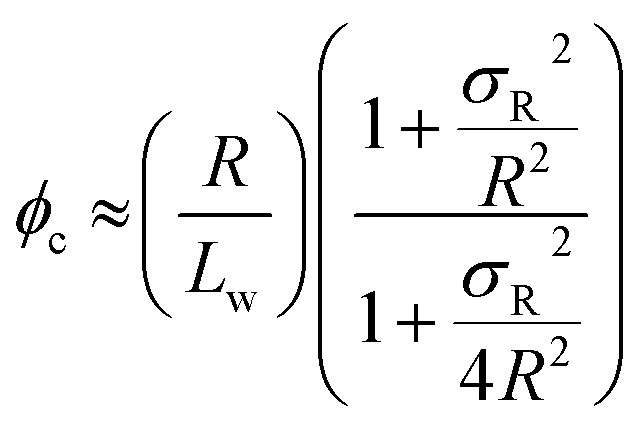
where *L*_w_ is the weight-average rod length, *R* is the number-average rod cross-sectional radius and *σ*_R_ is the standard deviation of the rod cross-sectional radius. As noted by Chatterjee, for populations of rods with narrow width polydispersities, relatively high aspect ratios, and uncorrelated variations in the widths and lengths, the percolation threshold is governed by the ratio of the number-average radius to the weight-average (rod) length. As noted above, the average worm cross-sectional radius *R* is well-defined, so *σ*_R_ tends to zero. Hence eqn (1) can be simplified to give:2
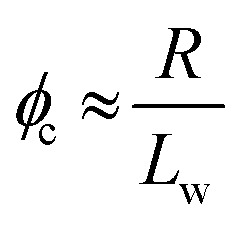
[*N.B.* It can be shown that the approximation made when deriving eqn (2) leads to a small systematic underestimation of *φ*_c_ (see ESI†)]. Otten and co-workers drew similar conclusions to that of Chatterjee using a somewhat different mathematical approach.^37^

**Scheme 1:**
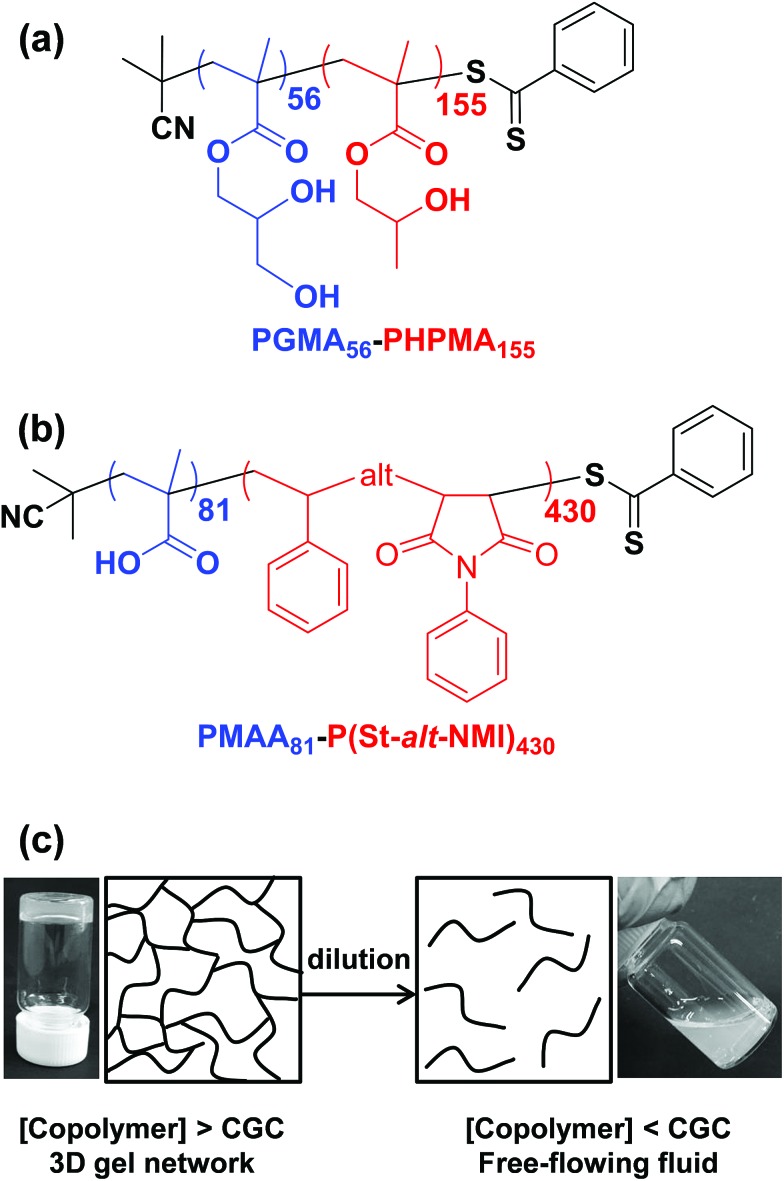
Chemical structures for (a) poly(glycerol monomethacrylate)-*block*-poly(2-hydroxypropyl methacrylate) (PGMA–PHPMA) diblock copolymers prepared by RAFT aqueous dispersion polymerization and (b) poly(methacrylic acid)-*block*-poly(styrene-*alt-N*-phenylmaleimide) copolymers prepared by RAFT dispersion polymerization in a 50/50% w/w ethanol/1,4-dioxane mixture. (c) Schematic cartoon illustrating formation of a continuous 3D network of worms above the critical gelation concentration (CGC) owing to multiple inter-worm contacts. In contrast, these inter-worm contacts are broken on dilution below the CGC, resulting in a free-flowing dispersion rather than a gel.

We postulated that the percolation threshold required for the formation of an extended 3D network of inter-connected electrically conductive rods randomly dispersed in an insulating matrix to produce macroscopic electrical conductivity^38^,^39^ should be equivalent to that required for formation of a macroscopic physical gel by a colloidal dispersion of rods. Herein, we evaluate to what extent eqn (2) provides a useful description of the gelation behavior observed for two examples of diblock copolymer worms.^36^,^37^ For this approach to be valid, gelation should occur as a result of multiple inter-worm contacts (see Scheme 1), which would provide an alternative gelation mechanism to the inter-worm entanglements model previously (and correctly) invoked for surfactant worms. The two diblock copolymer systems studied herein were chosen because they represent relatively long, highly flexible worms^25^ and relatively short, stiff worms, respectively.^40^ Thus they represent two limiting copolymer morphologies for which contrasting experimental data might be anticipated.

## Results and discussion

Initially, we sought literature data for colloidal dispersions of rigid rods to support our hypothesis. Recently, Nordenström *et al.* reported an interesting study of the aqueous gelation behavior of various cellulosic nanorods of varying dimensions and surface charge.^41^ More specifically, a series of six cellulose nanorods were prepared with varying length/diameter ratios (or aspect ratios) and their (de)gelation behavior was characterized using dynamic light scattering (DLS). The critical volume fraction for gelation was shown to be inversely proportional to the aspect ratio. Furthermore, it was postulated that gelation was simply a result of multiple contacts with neighboring nanorods, which arrests their translational diffusion in solution. However, no specific link was made to the recent mathematical advances developed to describe the percolation behavior of polydisperse rods.^36^,^37^ Of the six types of cellulose nanorods reported by Nordenström *et al.*,^41^ the most relevant to the present study of neutral worms is that with the lowest surface charge, which had a mean length of 520 nm (determined by DLS) and a mean radius of 3.35 nm (measured by AFM studies). Using eqn (2), we calculate the theoretical percolation volume fraction, *φ*_c_, for such cellulosic nanorods to be 0.0064, which is in reasonably good agreement with the experimental *φ*_c_ of 0.0073 reported by Nordenström and co-workers.^41^ Thus our hypothesis of physical equivalence between the respective critical percolation thresholds required for solid-state electrical conductivity and physical gelation appears to have some merit.

**Fig. 1:**
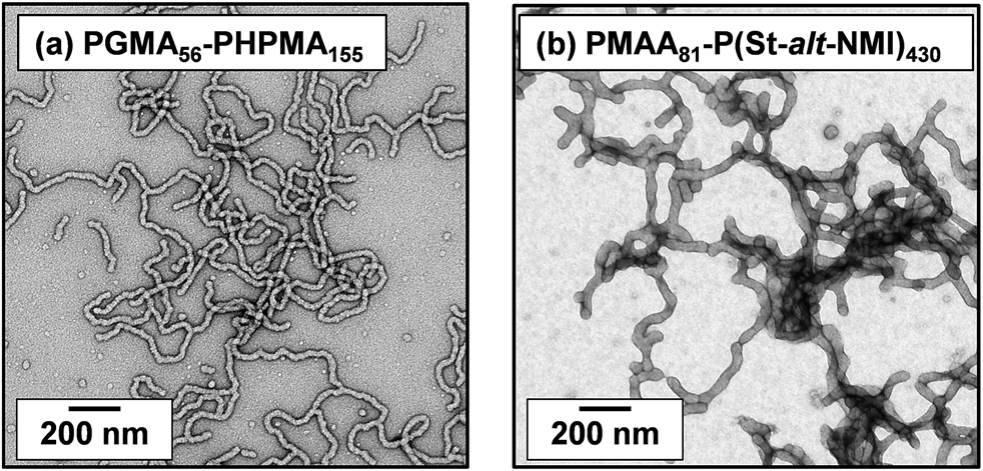
TEM images obtained for dry dispersions of (a) PGMA_56_–PHPMA_155_ and (b) PMAA_81_–P(St-*alt*-NMI)_430_ diblock copolymer worms prepared by PISA.

The poly(glycerol monomethacrylate)_56_–poly(2-hydroxypropyl methacrylate)_155_ [PGMA_56_–PHPMA_155_] and poly(methacrylic acid)_81_–poly(styrene-*alt-N*-phenylmaleimide)_430_ [PMAA_81_–P(St-*alt*-NMI)_430_] worm gels evaluated in this study were prepared using PISA as described by Blanazs *et al.*^22^ and Yang and co-workers^40^ respectively (see Scheme 1 for the relevant chemical structures). More specifically, the highly flexible PGMA_56_–PHPMA_155_ worms were synthesized *via* reversible addition–fragmentation chain transfer (RAFT) aqueous dispersion polymerization of 2-hydroxypropyl methacrylate (HPMA), and are clearly highly anisotropic as judged by transmission electron microscopy (TEM, see Fig. 1a). In contrast, the relatively short, stiff PMAA_81_–P(St-*alt*-NMI)_430_ worms were prepared by RAFT dispersion alternating copolymerization of styrene with *N*-phenylmaleimide using a 1 : 1 ethanol/1,4-dioxane mixture. These latter worms are much less anisotropic (see Fig. 1b). In both cases, the diblock copolymer chains possess relatively narrow molecular weight distributions as determined by gel permeation chromatography (GPC) and comparison to their respective macro-CTAs indicates high blocking efficiencies (see Fig. S1†). The mean aspect ratio (*i.e.* length/width ratio) for each type of worm can be determined using small-angle X-ray scattering (SAXS), as described below.^42^ TEM analysis confirms that the worm cross-sectional radius is well-defined in both cases. More specifically, the mean core radius, *r*_c_, for PGMA_56_–PHPMA_155_ and PMAA_81_–P(St-*alt*-NMI)_430_ is estimated to be 11.1 ± 1.3 and 19.2 ± 2.1 nm, respectively.

**Fig. 2:**
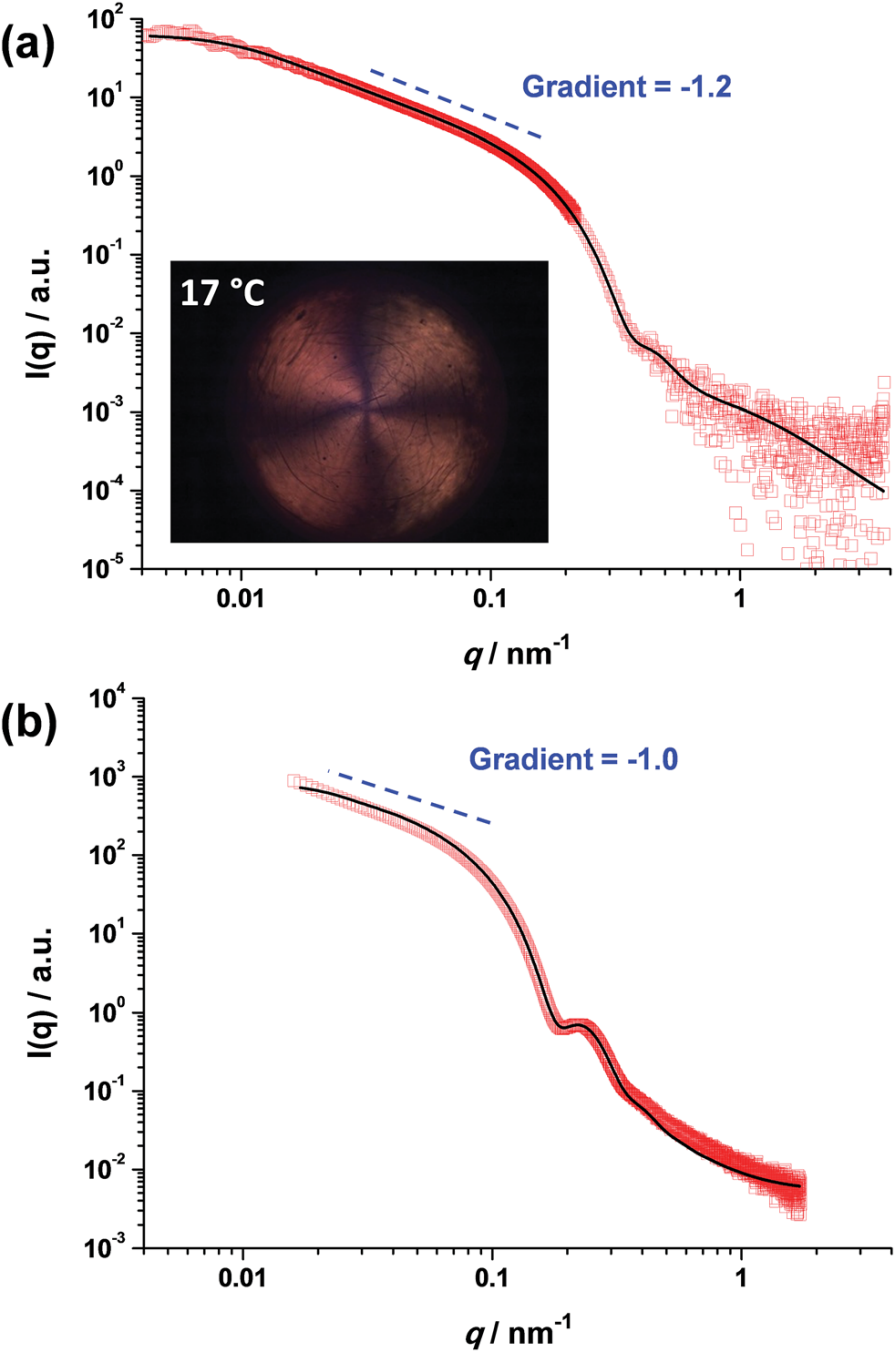
SAXS patterns recorded for 1.0% w/w dispersions of (a) PGMA_56_–PHPMA_155_ worms at 18 °C and (b) PMAA_81_–P(St-*alt*-NMI)_430_ worms at 20 °C. Inset in (a) shows a shear-induced polarized light image of the corresponding 5.0% w/w worm dispersion obtained at a maximum shear rate of 20 s^–1^. The Maltese cross observed is the distinctive signature for birefringence, indicating *in situ* worm alignment.^43^

In contrast, the worm contour length, *L*_w_, is clearly rather ill-defined. This is because such worms are formed *via* stochastic 1D fusion of multiple spheres during PISA.^44^ In principle, SAXS is a powerful technique for characterizing block copolymer nano-objects, not least because X-ray scattering is averaged over many millions of particles and hence much more statistically robust than TEM studies.^45^ Accordingly, SAXS patterns were recorded for the two worm dispersions at 1.0% w/w copolymer concentration, see Fig. 2. Both SAXS patterns exhibit a gradient of approximately –1 at low *q*, which is indicative of highly anisotropic rods (or worms). Fitting such patterns using an established worm model^46^ provides detailed and robust structural information, including the weight-average *L*_w_ and cross-sectional worm radius *R*. For example, SAXS indicates an *L*_w_ of approximately 1100 nm for PGMA_56_–PHPMA_155_ worms, with a corresponding core radius, *r*_c_, of 8.5 ± 0.9 nm (see Fig. 2a). However, the highly hydrated stabilizer chains also contribute to the overall effective worm dimensions. Given the mean DP of the PGMA_56_ chains, the thickness of this additional stabilizer layer is estimated to be 3.6 nm by SAXS analysis. Thus the effective worm cross-sectional radius, *R*, for these ‘hairy’ PGMA_56_–PHPMA_155_ worms is calculated to be 12.1 ± 0.9 nm (see ESI† for calculation details). In contrast, SAXS analysis of the PMAA_81_–P(St-*alt*-NMI)_430_ diblock copolymer worms suggests an *L*_w_ of approximately 296 nm (see Fig. 2b). These latter worms have an *r*_c_ value of 20.0 ± 2.7 nm and a stabilizer thickness of 6.6 nm, giving an overall *R* value of 26.6 ± 3.0 nm. Hence the mean aspect ratios (or *L*_w_/*R* values) for the PGMA_56_–PHPMA_155_ and PMAA_81_–P(St-*alt*-NMI)_430_ worms are 89 and 11, respectively. These strikingly different aspect ratios are useful in the context of the present study because they enable a more rigorous test of the percolation theory recently developed for polydisperse rods.^36^,^37^ Thus, according to eqn (2), the critical percolation volume fraction, *φ*_c_, required to form a 3D gel network comprising PGMA_56_–PHPMA_155_ worms is expected to be significantly lower than that required for gelation when using the PMAA_81_–P(St-*alt*-NMI)_430_ worms.

It is well-known that semi-concentrated dispersions of such PGMA–PHPMA worm gels exhibit thermoresponsive behavior, with degelation occurring on cooling below the critical gelation temperature (CGT) as a result of a worm-to-sphere transition.^20^,^25^ When applying percolation theory to such thermosensitive systems, it is important to determine the characteristic temperature that corresponds to long, linear worms (as opposed to branched worms or worm clusters). This is readily achieved using shear-induced polarized light imaging (SIPLI), as recently reported by Mykhaylyk and co-workers.^43^ Briefly, an aqueous worm dispersion is subjected to applied shear using an opto-rheometer, which enables simultaneous interrogation of the sample using polarized light. The appearance of a distinctive Maltese cross motif indicates shear-induced alignment of the highly anisotropic worms. If such experiments are performed as a function of temperature, the temperature at which the brightest Maltese cross is observed corresponds to the formation of the most linear (*i.e.* longest) worms. Such measurements are shown in the inset of Fig. 2a and S2† and indicate an optimum temperature of 17 °C, which is very close to that at which the SAXS studies were performed (18 °C). It is noteworthy that the PMAA_81_–P(St-*alt*-NMI)_430_ diblock copolymer worms do not exhibit such thermoresponsive behavior, so the temperature at which SAXS analysis is conducted is not particularly important in this case.

Utilizing the structural information provided by SAXS in combination with eqn (2), the theoretical critical volume fraction (*φ*_c_) required for the percolation threshold (and hence macroscopic gelation) is predicted to be 0.011 ± 0.001 and 0.090 ± 0.009 for the PGMA_56_–PHPMA_155_ and PMAA_81_–P(St-*alt*-NMI)_430_ worms, respectively. This approximate eight-fold difference simply reflects the substantial difference in aspect ratio for these two types of worms.

**Fig. 3:**
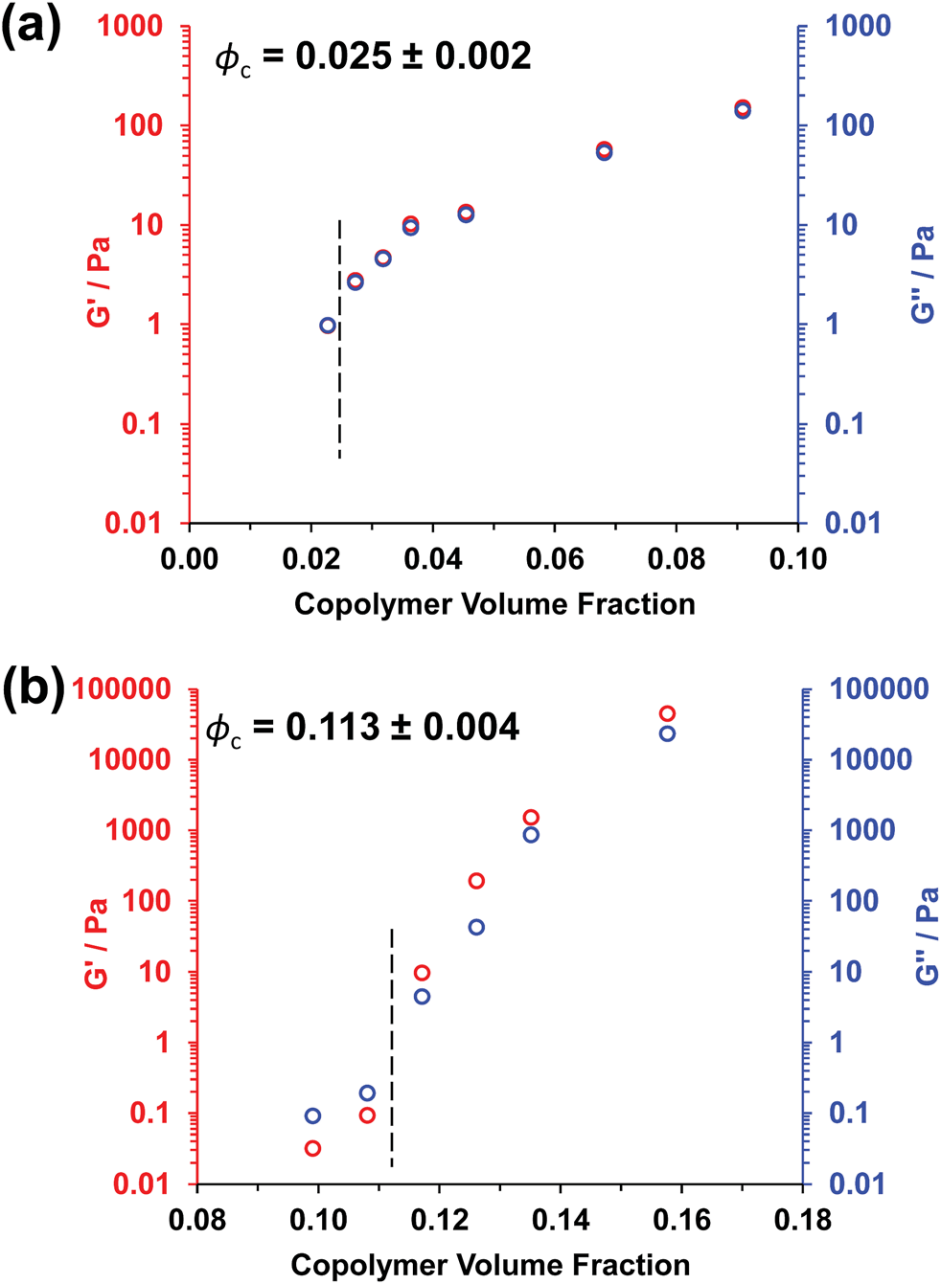
Oscillatory rheology data obtained for (a) PGMA_56_–PHPMA_155_ worms at 17 °C and (b) PMAA_81_–P(St-*alt*-NMI)_430_ worms at 20 °C at varying copolymer volume fractions. The critical gelation volume fraction (or *φ*_c_) is determined by the point of intersection of the loss modulus (*G*′′) and storage modulus (*G*′) curves.

Experimental *φ*_c_ values can be estimated from tube inversion tests, which were performed at ambient temperature (17–18 °C) for varying copolymer volume fractions (see Fig. S3† and related calculations). These observations indicated *φ*_c_ values of approximately 0.025 ± 0.002 and 0.121 ± 0.004 for the PGMA_56_–PHPMA_155_ and PMAA_81_–P(St-*alt*-NMI)_430_ worms, respectively. Very similar *φ*_c_ values (0.025 ± 0.002 and 0.113 ± 0.004) were obtained from oscillatory rheology, see Fig. 3. In this case, degelation is indicated by the point of intersection of the storage modulus (*G*′) and the loss modulus (*G*′′) curves, and these latter experiments are considered more reliable.

In polymer physics, the worm-like chain model is used to describe the behavior of semi-flexible polymers.^47^ For such worm-like chains, the Kuhn length is equal to twice the persistence length, where the latter parameter quantifies the chain stiffness. In principle, the behavior of the long, flexible diblock copolymer worms described in our study is analogous to that of an individual polymer chain.^48^ The Kuhn lengths derived from SAXS studies of both types of worms are included in Table 1. The Kuhn length for the short, stiff worms is simply equal to the weight-average worm contour length (*L*_w_). In contrast, the Kuhn length for the long, flexible worms is much lower than *L*_w_, which implies significant flexibility.

**Table 1:** Summary of weight-average worm length, cylindrical cross-sectional radius, aspect ratio, Kuhn length, theoretical and experimental volume fractions calculated for relatively flexible PGMA_56_–PHPMA_155_ worms and relatively stiff PMAA_81_–P(St-*alt*-NMI)_430_ worms

Anisotropic nanoparticle type	Weight-average worm length, *L*_w_/nm	Worm cross-sectional radius, *R*/nm	Aspect ratio (*L*_w_/*R*)	Kuhn length/nm	*φ*_c,_ theory^*a*^	*φ*_c,_ experiment^*b*^
PGMA_56_–PHPMA_155_ worms	1100	12.3	89	300	0.011	0.025
PMAA_81_–P(St-*alt*-NMI)_430_ worms	296	26.6	11	296	0.090	0.113

^*a*^Calculated using eqn (2).

^*b*^Determined by oscillatory rheology.

Clearly, there is some discrepancy between the theoretical and experimental *φ*_c_ values summarized in Table 1. However, percolation theory is derived assuming rigid rods, whereas the PGMA_56_–PHPMA_155_ worms clearly exhibit significant flexibility (see TEM images in Fig. 1). This necessarily reduces the effective weight-average worm length *L*_w_, which in turn leads to a higher *φ*_c_ value. Given this important caveat, the fair agreement observed between the experimental and theoretical *φ*_c_ values supports our hypothesis that such worm gels form a 3D network simply *via* multiple contacts between neighbouring worms. In contrast, the PMAA_81_–P(St-*alt*-NMI)_430_ worms are much stiffer (the glass transition temperature for the core-forming P(St-*alt*-NMI)_430_ block is around 208 °C.^40^ Thus, better agreement between experimental and theoretical *φ*_c_ values is expected, and indeed observed.

From Table 1, the theoretical *φ*_c_ for stiff worms is approximately eight times greater than that for flexible worms. In contrast, the corresponding experimental *φ*_c_ ratio is approximately four. This suggests that eqn (2) can be rewritten as:3
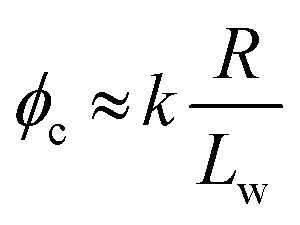
where the proportionality constant *k* varies by at least a factor of two depending on the degree of worm flexibility. It is perhaps also worth emphasizing here that, given the worm dimensions indicated by SAXS studies, the ‘worm entanglements’ mechanism invoked to account for the gelation of surfactant worms does not appear to account satisfactorily for the physical gelation observed for these much less anisotropic diblock copolymer worms.

## Conclusions

In summary, recent advances in percolation theory for polydisperse rods provide an improved understanding of the gelation behavior exhibited by diblock copolymer worms. Combined with experimental data, this suggests that a 3D gel network forms primarily *via* multiple contacts between neighbouring worms, rather than as a result of worm entanglements. In view of the growing number of studies utilizing worm-based hydrogels for various biomedical applications,^25^,^49^–^58^ this new physical insight is likely to be important for the design of next-generation diblock copolymer worm gels, as well as the growing literature on block copolymer rods.^58^–^62^ Indeed, it seems likely that our findings are also relevant to the growing literature on supramolecular gels composed of amphiphilic small molecules^63^–^71^ as well as hydrogels based on cellulose and silica nanorods.^41^,^72^–^77^

## Conflicts of interest

There are no conflicts to declare.
